# ORRM5, an RNA recognition motif-containing protein, has a unique effect on mitochondrial RNA editing

**DOI:** 10.1093/jxb/erx139

**Published:** 2017-05-26

**Authors:** Xiaowen Shi, Benoit Castandet, Arnaud Germain, Maureen R Hanson, Stéphane Bentolila

**Affiliations:** 1 Department of Molecular Biology and Genetics, Cornell University, Ithaca, NY, USA; 2 Boyce Thompson Institute, Cornell University, Ithaca, NY, USA

**Keywords:** Glycine-rich, mitochondria, plant development, plant editosome, plant stress response, RNA editing

## Abstract

Plants have an RNA editing mechanism that prevents deleterious organelle mutations from resulting in impaired proteins. A typical flowering plant modifies about 40 cytidines in chloroplast transcripts and many hundreds of cytidines in mitochondrial transcripts. The plant editosome, the molecular machinery responsible for this process, contains members of several protein families, including the organelle RNA recognition motif (ORRM)-containing family. ORRM1 and ORRM6 are chloroplast editing factors, while ORRM2, ORRM3, and ORRM4 are mitochondrial editing factors. Here we report the identification of organelle RRM protein 5 (ORRM5) as a mitochondrial editing factor with a unique mode of action. Unlike other ORRM editing factors, the absence of ORRM5 in *orrm5* mutant plants results in an increase of the editing extent in 14% of the mitochondrial sites surveyed. The *orrm5* mutant also exhibits a reduced splicing efficiency of the first *nad5* intron and slower growth and delayed flowering time. ORRM5 contains an RNA recognition motif (RRM) and a glycine-rich domain at the C terminus. The RRM provides the editing activity of ORRM5 and is able to complement the splicing but not the morphological defects.

## Teaser

Mutants lacking ORRM5, a mitochondrial protein carrying an RRM and glycine-rich domains, exhibit slower growth, delayed flowering, increased mitochondrial RNA editing extent, and reduced splicing of a *nad5* intron.

## Introduction

The RNA recognition motif (RRM) is a conserved ~80 amino acid motif that binds to RNA molecules with a wide range of specificities and affinities (Kenan *et al.*, 1991). As one of the most abundant protein motifs in eukaryotes, the RRM is involved in various processes of RNA metabolism, and also participates in plant stress responses and developmental processes (Maris *et al.*, 2005; Lorković, 2009). The numerous biological functions of RRM-containing proteins are likely due to the structural versatility of the RRM interactions, as well as the presence of variable auxiliary motifs.

Plant organelle-targeted RRM proteins have been shown to function in a variety of RNA processes, such as RNA splicing, RNA editing and RNA stability (Schmitz-Linneweber *et al.*, 2006; Ruwe *et al.*, 2011; Shi *et al.*, 2016*a*; Sun *et al.*, 2016). Studies from several groups have demonstrated that plant organelle-localized RRM proteins participate also in plant development and/or stress responses. For instance, *orrm4* mutants exhibited delayed growth and late flowering (Shi *et al.*, 2016*b*). CP29A and CP31A, members of the chloroplast ribonucleoprotein (cpRNP) family, influence multiple chloroplast RNA processes, including RNA stability, mRNA and rRNA processing under cold stress conditions (Tillich *et al.*, 2009; Kupsch *et al.*, 2012). Another member of the cpRNP family, CP33A, contributes to RNA stability, and is required for chloroplast biogenesis and plant development. *cp33a* null mutants survived only when provided with an external carbon source and exhibited aberrant leaf development (Teubner *et al.*, 2016). A plastid protein named RNA-binding domain-containing protein 1 (RBD1) is involved in chilling tolerance in Arabidopsis, presumably by regulating 23S rRNA processing (Wang *et al.*, 2016). However, the molecular mechanism underlying the participation of RRM proteins in both RNA-related processes and plant development and/or stress responses is still elusive.

Organelle RNA recognition motif-containing protein 1 (ORRM1) is essential for the post-transcriptional cytidine (C)-to-uridine (U) RNA editing in the Arabidopsis chloroplast (Sun *et al.*, 2013). By analysing ORRM family members, we subsequently identified ORRM2, ORRM3, and ORRM4 as mitochondrial RNA editing factors whereas ORRM6 is a chloroplast RNA editing factor (Shi *et al.*, 2015, 2016*b*, Hackett *et al.*, 2017). Unlike ORRM1, which carries a truncated RNA editing factor interacting protein (RIP)–RIP motif at its N terminus and an RRM at its C terminus, ORRM2 and ORRM6 carry only an RRM, while ORRM3 and ORRM4 each contain an N-terminal RRM and a C-terminal glycine-rich (GR) motif (Sun *et al.*, 2013; Shi *et al.*, 2015, 2016*b*; Hackett *et al.*, 2017). The RRM in ORRM1, ORRM3, or ORRM4 is sufficient for the protein’s function in editing, whereas the auxiliary RIP or GR motif is responsible for mediating its interaction with other *trans*-acting factors in the RNA editing complex (Sun *et al.*, 2013, 2016; Shi *et al.*, 2015, 2016*b*).

While investigating the function of the ORRM family members through analysis of T-DNA insertional mutants, we found that the altered expression of a gene in the ORRM family, encoded by At4g13850, causes a delayed growth and late flowering phenotype. We named this protein organelle RNA recognition motif-containing protein 5 (ORRM5). In order to investigate the cause of the morphological defects in the *orrm5* mutants, we examined the effect of the *ORRM5* mutations on RNA splicing, the abundance of certain transcripts, and RNA editing. *ORRM5* mutations cause reduction of *cis*-splicing efficiency of the first intron of the mitochondrial *nad5* transcript. Mutations in *ORRM5* result in decreased editing efficiency at 18 mitochondrial C targets, while editing extents increased at 79 mitochondrial sites compared with the wild-type editing level. Therefore, the absence of ORRM5 results in an increase of editing extent in 14% of the mitochondrial sites surveyed. ORRM5 is the first editing factor reported to have such an inhibitory impact on plant organelle editing. Interaction data presented in this report suggest the hypothesis that the effect of ORRM5 on editing might be mediated through the sequestration of other ORRM mitochondrial editing factors.

## Materials and methods

### Plant material and morphological analysis

The Arabidopsis T-DNA insertion lines SALK_059714C (*orrm5-2*) and SALK_135802C (*orrm5-3*) in the *ORRM5* gene were ordered from the Arabidopsis Biological Resource Center (ABRC; https://abrc.osu.edu/). After 3 days of stratification, seeds from the mutant line were planted in soil and grown in a growth room (14 h of light/10 h of dark) at 26 °C. Plants were genotyped by PCR with BioMix Red (Bioline) using primers listed in Supplementary Table S1 at *JXB* online. The PCR products were sequenced at Cornell University Life Sciences Core Laboratories Center. Leaves were collected from 5-week-old plants for strand- and transcript-specific RNA-seq (STS-PCRseq). The *ORRM5* expression level was measured by quantitative RT-PCR. All the primers used are listed in Supplementary Table S1.

The fresh weight of plants grown at 14 h of light per day was measured 26, 34, 36 and 38 days after planting. We recorded the number of days it took for visible flower buds to show in the center of the rosette, for the inflorescence stem of the plant to reach 1 cm in height, and for its first flower to open. Information regarding the fresh weight and the number of total leaves of the mutant plants, transgenic lines *versus* the controls, was recorded when the first flower bloomed.

### Generation of transgenic plants

The coding sequence of *ORRM5* was reverse-transcribed with SuperScript® III Reverse Transcriptase (Life Technologies) from RNA extracted from wild-type Arabidopsis Columbia using PureLink® RNA Mini Kit (Life Technologies), and then cloned into a PCR8/GW/TOPO vector. The N-terminal *ORRM5* was amplified from the reverse-transcribed *ORRM5* coding sequence with primer pair ORRM5-F and ORRM5-345RTAG. Primers used are listed in Supplementary Table S1. The fragments were subsequently shuffled into a modified pBI121 vector using LR Clonase II. 35S-ORRM5 and 35S-nORRM5 in the pBI121 vector were transformed into *Agrobacterium tumefaciens* GV3101. Floral dip transformation of homozygous *orrm5* mutant plants was performed as described in Zhang *et al.* (2006). Plants were sprayed with Basta twice on soil for selection. The presence of the transgene and the homozygosity of the *orrm5* mutant allele were verified by PCR reactions using primers listed in Supplementary Table S1. Leaves from 4- to 6-week-old transgenic plants were collected for further analysis. 35S-nORRM4 and 35S-cORRM4 transgenic lines were from a previous study (Shi *et al.*, 2016*b*).

### Use of STS-PCRseq method to assay editing extent

The STS-PCRseq technique was discussed in detail in a previous study (Bentolila *et al.*, 2013). We amplified all transcripts encoding either plastid or mitochondrial genes with organellar transcript-specific primers from the mutant tissue and the controls. Primers used are listed as in Bentolila *et al.* (2013). The RT-PCR products were mixed in equimolar ratio, shared by sonication, and then used as templates to produce an Illumina TruSeq DNA Library. All the samples in this study were pooled in one sequencing lane of an Illumina HiSeq 2500 instrument. The data we obtained were processed according to the guideline provided in a previous study (Bentolila *et al.*, 2013). All the read numbers for each editing site determined in this study are in the Supplementary Dataset S1. Subsequent statistical analysis was mostly similar to the one performed in three previous studies (Bentolila *et al.*, 2013; Shi *et al.*, 2015, 2016*b*). The test for a significant difference in editing extent between the mutant plants and the wild-type plants varies slightly in the present study. In previous reports the data were pooled between biological replicates, either wild-type plants or mutant plants; the difference in editing extent between wild-type and mutant was then tested by a chi-square test with one degree of freedom, one test for each editing site. In this study we did not pool the reads between biological replicates. To declare a significant difference in editing extent between a wild-type and a mutant, each biological replicate had to satisfy the chi-square test, so four chi-square tests had to be positive, instead of one. Because of repetitive testing, we chose a nominal error rate of *P*<1.6 × 10^–6^ to achieve the desired family error rate of *P*<1 × 10^–3^ when analysing 612 sites (36 plastid sites + 576 mitochondrial sites). In addition to this chi-square test requirement, a site was declared significantly reduced (increased) in its editing extent in the *orrm5* mutant if the reduction (increase) compared with the wild-type plant was ≥0.1. This new methodology is more conservative and results in less difference in editing extent being called. For the transgenic plants, a site was declared significantly affected when the chi-square test between the transgenic plant and the corresponding mutant transformed to obtain the transgenic (T5, T6, T9, T10 *vs orrm5-2*-1 and *orrm5-2*-2; T7, T8, T11, T12 *vs orrm5-3*-1 and *orrm5-3*-2) satisfied the threshold requirement (*P*<1.6 × 10^–6^). In addition, the absolute value of the variation in editing extent had to be ≥0.1 (≥0.1 for an increase and ≤–0.1 for a decrease).

### Real-time quantitative RT-PCR conditions and analysis

The real-time qRT-PCR was performed as described in a previous study (Shi *et al.*, 2015). Primers used in the reaction are listed in Supplementary Table S1. The splicing efficiency was estimated by the level of expression of two amplicons, one specific to the spliced transcript and amplified with the primers nad5-ex1F and nad5-ex2R, one specific to the unspliced transcript and amplified with the primers nad5-ex1F and nad5-int1R (see Supplementary Table S1). Splicing efficiency was determined as: (nad5-ex1F, nad5-ex2R)/[(nad5-ex1F, nad5-ex2R)+(nad5-ex1F, nad5-int1R)]

### Chloroseq use and application to STS-PCRseq data

The STS-PCRseq sequencing reads were aligned to the Arabidopsis chloroplast and mitochondrial genomes (TAIR10 version) using Tophat2 (Trapnell *et al.*, 2010). The aligned reads were then used as input for ChloroSeq splicing analysis (option-a 2) (Castandet *et al.*, 2016) using custom annotations files containing the introns and splice coordinates. The annotation files used can be accessed online at https://github.com/BenoitCastandet/chloroseq/tree/master/TAIR10_ChrM_files.

### Yeast-two hybrid assay

The *ORRM5* coding sequence with its predicted transit peptide removed (amino acids 1–32) was amplified from the reverse-transcribed *ORRM5* cDNA clone with primer pairs ORRM5-97F and ORRM5-R listed in Supplementary Table S1. The PCR product was integrated into PCR8/GW/TOPO vectors and then shuffled to pGADT7GW or pGBKT7GW vectors (Horák *et al.*, 2008). All other constructs used in the Y2H assay were from previous studies (Bentolila *et al.*, 2012; Sun *et al.*, 2013, 2015; Shi *et al.*, 2015, 2016*b*).

Two mating types of yeast strain PJ69-4, a and α, were transformed with the constructs above as described in Gietz *et al.* (1995). Double transformants were produced by mating single transformants of mating type a to mating type α, cultured in leucine- and tryptophan-dropout media (Clontech), and subsequently diluted with sterile water to 1 × 10^6^ and 1 × 10^5^ cells ml^–1^; 10 µl of each dilution was spotted on leucine-, tryptophan-, adenine- and histidine-dropout media plates. Yeasts transformed with empty vectors were used as negative controls to detect auto-activation. Data were collected from 2 d to 5 d after spotting.

### Bimolecular fluorescence complementation assay

The coding sequence of *ORRM5*, without the stop codon, was amplified from the full-length cDNAs as described above, and cloned into PCR8/GW/TOPO vectors (Invitrogen). The fragment was then shuffled into XNGW and XCGW vectors (Ohashi-Ito and Bergmann, 2006) by LR reactions. *ORRM3*, *ORRM4*, and *RIP1* constructs were from previous studies. All the primers used are listed in Supplementary Table S1. Final vectors were validated by sequencing and transformed into *Agrobacterium tumefaciens* GV3101. Agrobacterium infiltration and confocal imaging were as described in (Shi *et al.*, 2016*b*).

### RNA blots

RNA gel blot analysis was performed as described in Germain *et al.* (2011). Primers used to make the probes are listed in Supplementary Table S1.

## Results

### ORRM5 mutations lead to delayed growth and late flowering

**Fig. 1.**
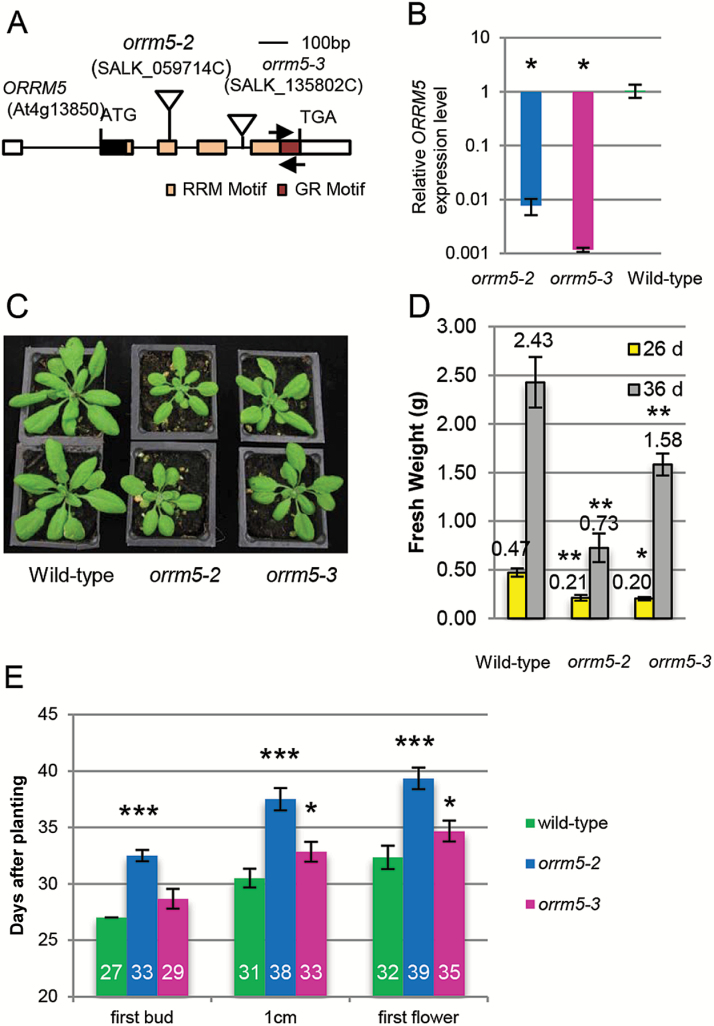
*ORRM5* mutations lead to delayed growth and late flowering. (A) Gene structure of *ORRM5*. Triangle indicates the locus of the T-DNA insertion. Primers used for the qRT-PCR are represented by arrows. (B) Relative *ORRM5* expression level measured by qRT-PCR. *ORRM5* expression is reduced to an undetectable level in the two *orrm5* mutant lines compared with the wild-type plants (*n*=3). (C) Plant growth phenotype of wild-type Arabidopsis (left), *orrm5-2* (middle), and *orrm5-3* (right) homozygous mutants grown at 14 h of light per day for 26 d. (D) Fresh weight of *orrm5* mutants and wild-type plants grown at 14 h of light per day for 26 and 36 d (*n*=5). (E) Days taken for plants to reach these developmental stages. First bud, days until visible flower buds in the center of the rosette; 1 cm, days until inflorescence stems reached 1 cm in height; first flower, days until first open flower (*n*=6). Student’s *t*-test: *P<0.05, **P<0.01, ***P<0.001 in comparison with the wild-type. In (B, D, E) values represent mean±SD.

In order to characterize the function of ORRM5, we obtained two T-DNA insertional mutant lines from the ABRC. The *orrm5-2* mutant (SALK_059714C) contains a T-DNA insertion in its second exon, whereas the *orrm5-3* mutant (SALK_135802C) carries an insertion in its fourth intron (Fig. 1A). Both mutant lines, in the Columbia background, are knockout mutants since the *ORRM5* expression level is decreased to an undetectable level as measured by qRT-PCR (Fig. 1B). The level of *ORRM5* expression in *orrm5-2* mutants is not significantly different from that in *orrm5-3* mutants (Student’s *t*-test; *P*>0.05) (Fig. 1B). We were able to retrieve wild-type siblings (*ORRM5-3*+/+) and *orrm5-3* mutant plants (*orrm5-3*–/–) from the *orrm5-3* mutant population while the *orrm5-2* mutant population did not segregate and exhibited only homozygous mutants (*orrm5-2*–/–). *ORRM5* mutations result in a delayed growth and late flowering phenotype compared with wild-type Arabidopsis plants (Col-0) (Fig. 1C–E). As shown in Fig. 1C, D, *orrm5-2* and *orrm5-3* homozygous mutants grew slower and had lower fresh weight than the wild-type plants under the long-day conditions (14 h light day^–1^). In order to examine the flowering phenotype of the *orrm5* mutants, we assayed three flowering time-related traits. The results indicate that *orrm5-2* mutants required ~7 days more on average for their first flower bud to become visible in the center of the rosette, for their inflorescence stems to reach 1 cm in height, and for their first flower to open compared with wild-type plants (Fig. 1E). For *orrm5-3* mutants, ~2 days more were required on average for the plants to reach these stages compared with wild-type plants (Fig. 1E).

### *ORRM5* mutations cause changes in mitochondrial RNA editing extents

**Fig. 2.**
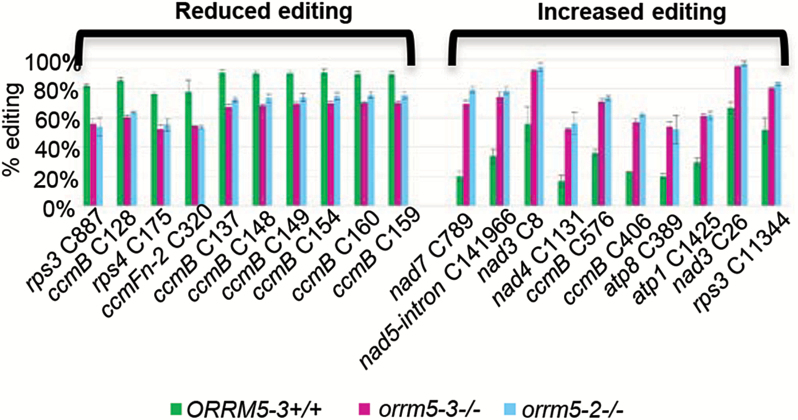
*ORRM5* mutations cause mitochondrial editing defects. Ten sites that experienced a significant decrease of editing extent (Δ ≥10%) upon *ORRM5* mutations (left), and ten sites that showed a significant increase of editing extent (Δ ≥10%) in the *orrm5* mutants (right). *ORRM5-3*+/+, wild-type siblings of *orrm5-3* mutants; *orrm5-3*–/–, *orrm5-3* homozygous mutants; *orrm5-2*–/–, *orrm5-2* homozygous mutants (*n*=2). Editing sites are displayed according to the difference between the wild-type and the mutants, from highest to lowest. Values represent mean±SD.

ORRM5 is located in the mitochondrion, according to proteomic and genetic analysis (Kruft *et al.*, 2001; Vermel *et al.*, 2002; Heazlewood *et al.*, 2004). In order to characterize the role of ORRM5 in mitochondrial RNA editing, we examined the effect of *ORRM5* mutations on editing extents using an approach named strand- and transcript-specific RNA-seq (STS-PCRseq) (Bentolila *et al.*, 2013). Two biological replicates were assayed for each sample, the two mutant plants *orrm5-2* and *orrm5-3*, and the wild-type siblings of *ORRM5-3*. Mutations in *ORRM5* cause decreased editing efficiency at 18 (30) mitochondrial sites in *orrm5-2* (*orrm5-3*), but lead to increased editing extents at 100 (86) mitochondrial C targets (*P*<1.6 × 10^–6^, Δ*orrm5-3*≥10%, Δ*orrm5-2*≥10%). When taking into account the common sites affected in both *orrm5-2* and *orrm5-3*, *ORRM5* mutations result in RNA editing increases at 79 mitochondrial sites, or 14% of the total mitochondrial sites assayed in this study. Eighteen mitochondrial sites or 3% of the total mitochondrial sites display a decrease of editing extent in both mutants. Figure 2 illustrates ten editing sites that experience significantly decreased editing extents as well as ten sites that show significantly increased editing efficiency in *ORRM5* mutants.

In order to examine the distribution of the affected C targets on different transcripts, we calculated the percentage of affected edited sites per transcript in the *orrm5* mutants based on where they are located and on the complex encoded by the affected transcripts (see Supplementary Fig. S1). The effect of *ORRM5* mutations on RNA editing was distributed among 24 mitochondrial transcripts (Supplementary Fig. S1A).

For the majority of transcripts, the percentage of affected sites per transcript is between 10% and 40%. We categorized the sites that show editing defects upon *ORRM5* mutations into two subgroups: the group that experiences reduced editing and the group that exhibits increased editing in the *orrm5* mutants (see Supplementary Fig. S1B, C). Sites that experience a reduction of editing extents are distributed on five transcripts, while sites with increased editing extents were more evenly distributed on 23 transcripts.

Transcripts encoding complex V, complex III, complex IV, and complex I subunits exhibit sites that experience only increased editing rather than reduced editing extent in *orrm5* mutants (see Supplementary Fig. S1). Transcripts encoding the cytochrome *c* biogenesis complex are rather unique in their response to the *orrm5* mutation since 11% of their editing sites show a reduction when compared with the wild-type (Supplementary Fig. S1). *rps3* and *rps4* are the only other transcripts experiencing a decrease in editing extent in the *orrm5* mutants.

The effect of *ORRM5* mutations on different transcripts exhibits a variety of patterns. For example, editing extents of all the C targets on the *nad3* transcript are increased in the *orrm5* mutants (see Supplementary Figs S1C and S2A). However, the alteration of editing extents on the *nad3* transcript shows some site specificity, as the effect varies from ~15% to ~70% (Supplementary Fig. S2A). On some transcripts, the effect of *ORRM5* mutation can be either inhibitory or stimulatory, depending on the site. For instance, on the *rps4* transcript, the absence of *ORRM5* expression causes a reduction of editing at three C targets, an increase of editing at two C targets, and leaves the remaining 15 sites unaffected (see Supplementary Fig. S2B). Like *rps4*, *ccmB*, *ccmC*, and *rps3* transcripts carry sites that show a reduction of editing extent in the *orrm5* mutant while other sites experience an increase of editing extent (Supplementary Fig. S1B, C).

### Stable expression of *ORRM5* complements the editing defects in the orrm5 mutants

**Fig. 3.**
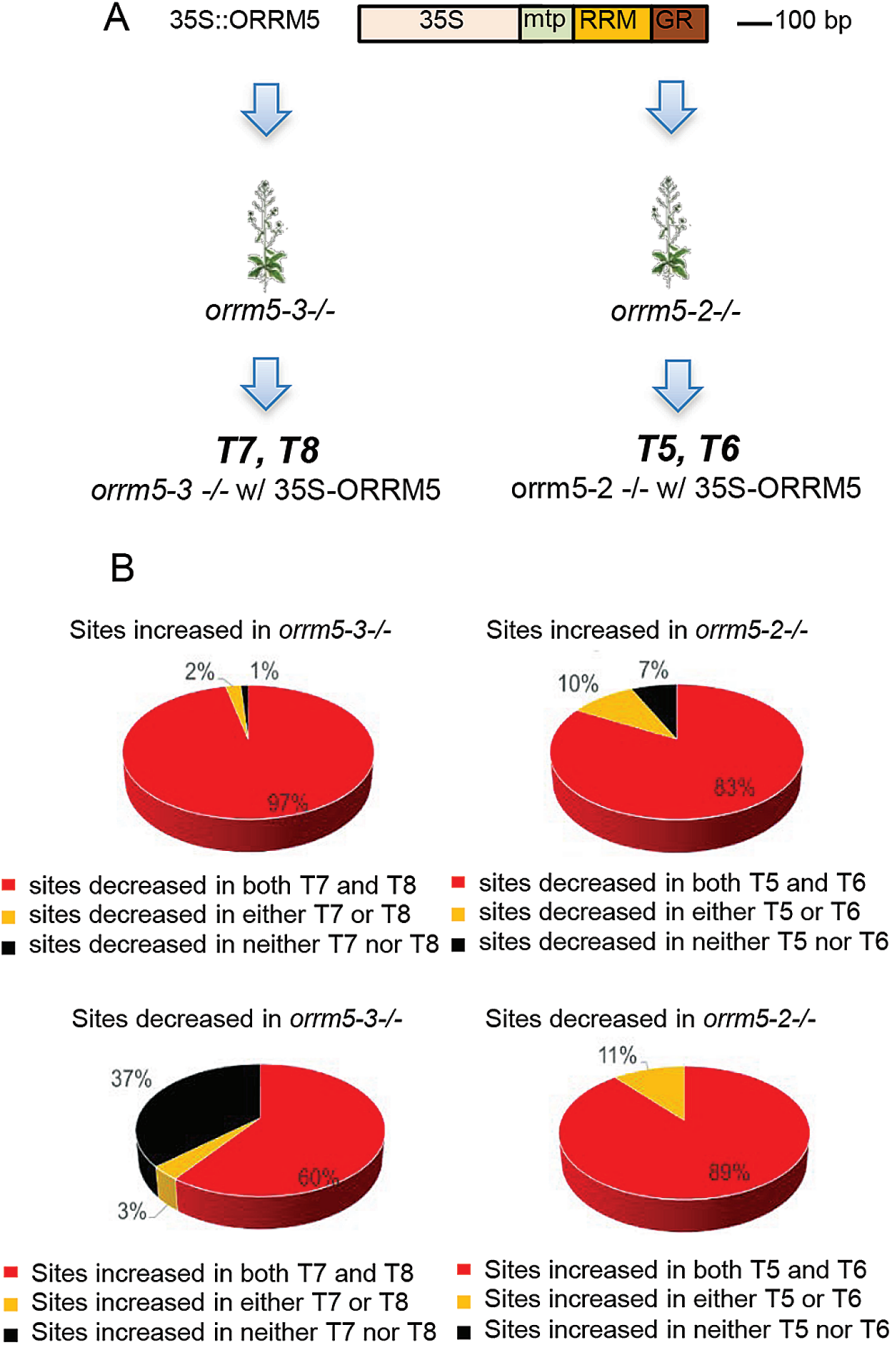
Complementation of mitochondrial editing defects in *orrm5* mutants by expressing *ORRM5*. (A) Constructs used for generation of transgenic plants. (B) Stable expression of *ORRM5* complements the majority of the editing defects caused by *ORRM5* mutations. Editing extents were measured by STS-PCRseq in the transgenic plants. Pie charts represent the proportion of the sites affected in the mutants that are affected in both (red), only one (orange), or neither (black) transgenic plants.

To test whether the editing defects in the *orrm5* mutants are truly caused by *ORRM5* mutations, we transformed *orrm5-2* or *orrm5-3* mutant plants with a construct expressing the coding sequence of *ORRM5* under the control of a 35S promoter, as shown in Fig. 3A. We performed genotyping of plants that survived in the Basta selection to verify the homozygosity of the T-DNA insertion allele and the presence of the *ORRM5* transgene. Afterwards, we collected tissue from two independent transgenic plants from the T_0_ generation, and assayed their editing extents by STS-PCRseq. We analysed the sites that were affected in *orrm5-2* and *orrm5-3* and determined whether their editing extents were significantly changed in the transgenic plants when compared with the respective mutants, T5 and T6 *vs orrm5-2* and T7 and T8 *vs orrm5-3* (Fig. 3A). Sites showing an increase of editing extent in the mutants relative to the wild-type are expected to exhibit a decrease of editing extent in the transgenic plants, while sites showing a decrease of editing extent in the mutants should show an increase in the transgenic plants. The vast majority of sites with an increase in the mutants display a significant decrease (*P*<1.6 × 10^–6^, |ΔT|≥10%) in the transgenic plants (upper panel, Fig. 3B). Among the 86 sites that show an increase in editing extent in *orrm5-3*, 83 sites or 97% show the expected decrease in both transgenic plants. This percentage reaches 99% when sites showing a decrease in only one transgenic plant are included. The same observation holds true for the sites showing an increase in *orrm5-2*, where 93% of these display a decrease in at least one transgenic plant (upper panel, Fig. 3B). We also analysed the response observed in the transgenic lines by defining a metric we call the complementation effect. This complementation effect normalizes the difference of editing extent between the transgenic and the mutant plants to the difference observed between the wild-type and the mutant plants (see Supplementary Fig. S3). Because *ORRM5* is under the control of the strong 35S promoter in the transgenic lines, the majority of the sites exhibit a transgressive response (complementation effect >1) in the transgenic lines, particularly T7 and T8, obtained by transforming *orrm5-3* mutant (Supplementary Fig. S3A).

While all the sites decreased in *orrm5-2* show an increase in at least one of the transgenic plants, this fraction drops to 63% for *orrm5-3*, presumably because of an inadequate level of expression of the transgene in T7 and T8 (Fig. 3B, lower panel). This result is also apparent when analysing the complementation effect of the sites; in T7 and T8 the majority of the sites fell below a complementation effect of 1, while in T5 and T6 the majority of the sites show a complementation effect >1 (see Supplementary Fig. S3B). When combining all the 234 sites affected, either increased or decreased, in both *orrm5-2* and *orrm5-3* mutants, 215 sites among those, or 92%, display the expected alteration of editing extent in the transgenic plants.

Numerous invariant mitochondrial sites that did not show significant change in their editing extent in the mutants when compared with the wild-type were significantly affected in the transgenic lines transformed with the full length *ORRM5*; 125 mitochondrial sites exhibited a significant alteration of their editing extent in T7 and T8 (compared with *orrm5-3*), and 69 mitochondrial sites underwent significant change of editing extent in T5 and T6 (compared with *orrm5-2*). Among these, the majority showed a decrease of editing extent when compared with the respective mutant plants, thereby supporting the inhibitory effect of ORRM5 on mitochondrial editing (see Supplemental Fig. S4).

### The N-terminal RRM of *ORRM5* can rescue most of the editing defects in the *orrm5* mutants

**Fig. 4.**
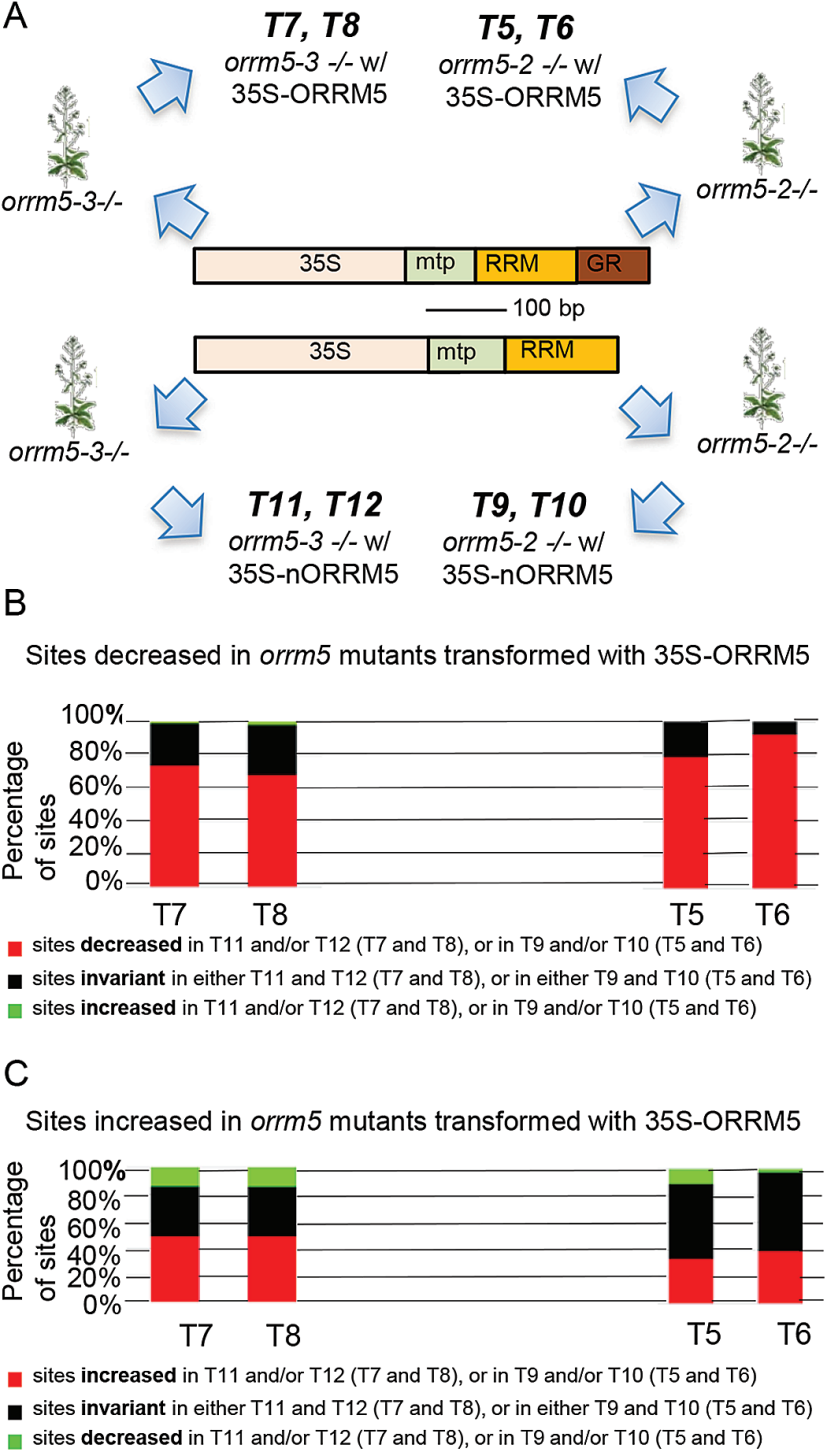
Proportion of the sites altered in full length transgenic plants that are decreased, invariant, or increased in transgenic plants transformed with the RRM containing part of *ORRM5*. (A) Constructs used for generation of transgenic plants. (B) Proportion of the sites decreased in full length transgenic plants that are decreased, invariant, or increased in the RRM transgenic plants. (C) Proportion of the sites increased in full length transgenic plants that are increased, invariant, or decreased in the RRM transgenic plants.

ORRM5 carries an RNA recognition motif (RRM) at its N terminus and a glycine-rich (GR) motif at its C terminus. In order to characterize the role of the RRM in RNA editing, we transformed *orrm5* mutants with a construct expressing the N-terminal RRM (amino acid 1–115) of *ORRM5* under the control of a 35S promoter but lacking the GR motif (Fig. 4A). Two independent transgenic plants surviving in the Basta selection from the T_0_ generation were selected, verified by genotyping, and analysed by STS-PCRseq. We first analysed the sites that showed a decrease in editing extent in the transgenic plants transformed with the full length *ORRM5* and determined how these sites behaved in the transgenic plants transformed with the RRM. The RRM was able to induce a decrease in editing extent in ~70% of the sites in the *orrm5-3* mutant and 85% of the sites in the *orrm5-2* mutant (Fig. 4B). On average, the RRM was able to cause a decrease in 77% of the sites that were decreased in editing extent in the transgenic plants transformed with the full length *ORRM5*.

In contrast, fewer of the sites that exhibited an increase in editing extent in transgenic plants transformed with the full-length *ORRM5* also exhibited increases in plants expressing the RRM only (Fig. 4C). Only 49% of these sites display an increase of editing extent in the *orrm5-3* mutant complemented with only the RRM, while even fewer sites experience an increase in the RRM-expressing *orrm5-2* mutant. The RRM was able to cause an increase of editing extent in only 43% of the set of sites that were increased in all four transgenic plants transformed with the full-length *ORRM5*. The effect of the RRM construct was even opposite to the one caused by the full length *ORRM5* in 14 % of the sites in *orrm5-3* transgenic plants (green bar, Fig. 4C). Interestingly, all these sites are located on the *rps4* transcript (see below).

**Fig. 5.**
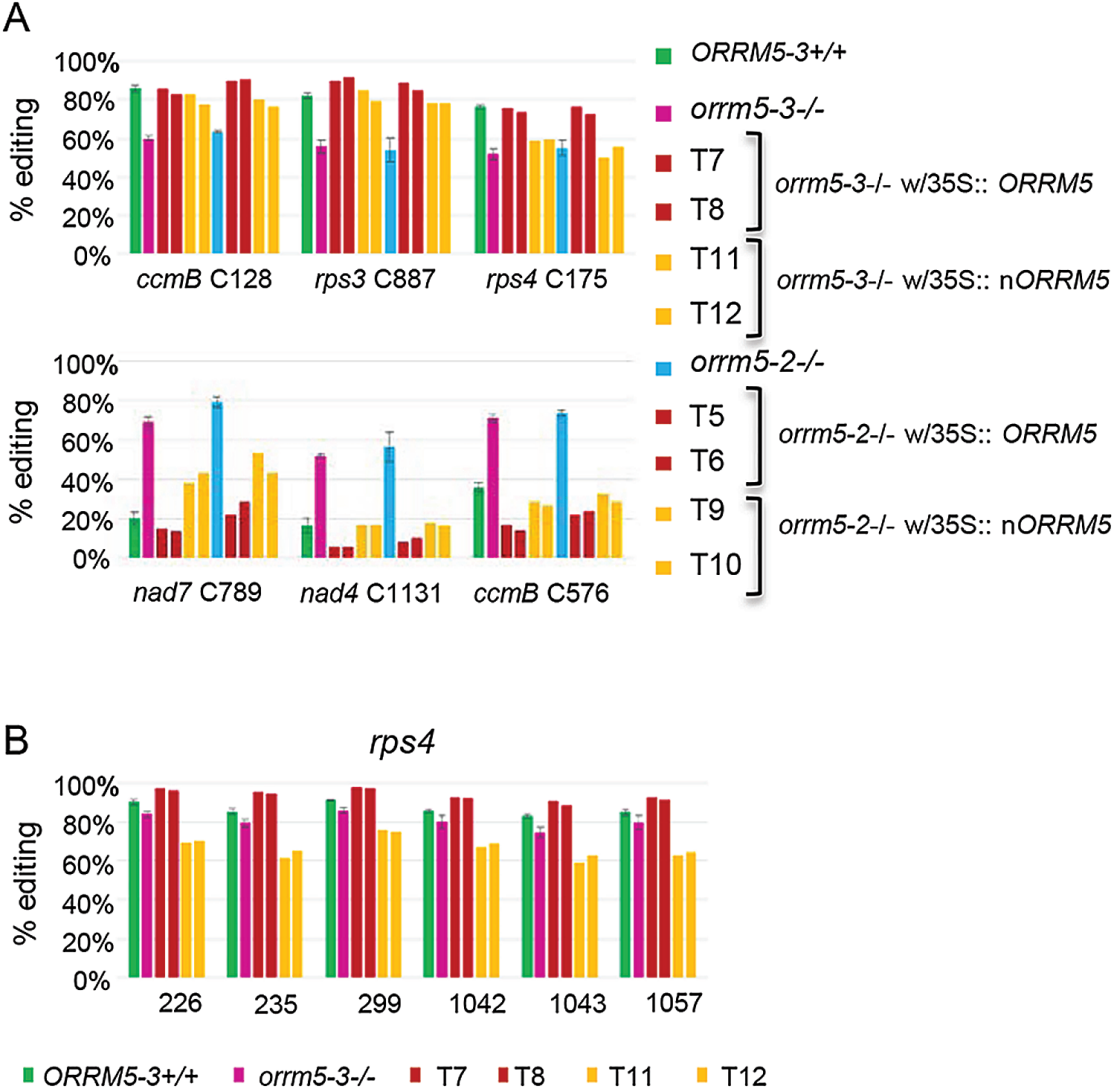
Complementation of mitochondrial editing defects in *orrm5* mutants by expressing *ORRM5* or the N-terminal RRM of *ORRM5*. (A) Stable expression of *ORRM5* or *nORRM5* (RRM) complements the editing defects caused by *ORRM5* mutations. Editing extents were measured by STS-PCRseq. T7 and T8: *orrm5-3*–/– w/35S:: *ORRM5*; T11 and T12: *orrm5-3*–/– w/35S:: *nORRM5*; T5 and T6: *orrm5-2*–/– w/35S:: *ORRM5*; T9 and T10: *orrm5-2*–/– w/35S:: n*ORRM5.* Top, the decrease of editing at sites *ccmB* C128, *rps3* C887, and *rps4* C175 in the *orrm5* mutants is complemented in the transgenic plants expressing *ORRM5* or *nORRM5*. Bottom, the increase of editing at sites *nad7* C789, *nad4* C1131, and *ccmB* C576 in the *orrm5* mutants is complemented in the transgenic plants expressing *ORRM5* or *nORRM5*. (B) The effects of RRM and full length *ORRM5* on the editing extent of *rps4* sites are in opposite direction in transgenic *orrm5-3* plants. Full length *ORRM5* increases the editing extent in *orrm5-3* transgenic plants T7 and T8, while the RRM decreases the editing extent in *orrm5-3* transgenic plants T11 and T12. Values represent mean±SD for *ORRM5-3*+/+, *orrm5-3*–/–, and *orrm5-2*–/–.

As an illustration of the different classes of mitochondrial editing sites we encountered during our analysis, we show the results of six mitochondrial C targets, *ccmB* C128, *rps3* C887, and *rps4* C175 with reduced editing extents, and *nad7* C789, *nad4* C1131, and *ccmB* C576 with increased editing extents in the *orrm5* mutants (Fig. 5A). The editing level at sites *ccmB* C128, *rps3* C887, and *rps4* C175 is complemented to the wild-type level or even higher in the transgenic lines expressing the full length *ORRM5*, while the RRM is able to increase the editing extents to the wild-type level or slightly lower for the *ccmB* C128 and *rps3* C887 sites (Fig. 5A, top). The RRM has no effect on the editing extent at the *rps4* C175 site in both *orrm5-3* (T11, T12) and *orrm5-2* (T9, T10) transgenic plants (Fig. 5A, top). The same trend is observed for sites exhibiting an increase of editing extent in the *orrm5* mutants with a more pronounced effect of the full length *ORRM5* than the RRM on the editing extent in the transgenic plants (Fig. 5A, bottom). At sites *nad4* C1131 and *ccmB* C576, both the full length *ORRM5* and the RRM reduce the editing extent in transgenic plants to the wild-type level or even lower (Fig. 5A, bottom). At the *nad7* C789 site, the full length *ORRM5* restores the editing extent to wild-type level or slightly higher, while the RRM significantly decreases the editing extent compared with the *orrm5* mutants, but does not decrease it to the wild-type level (Fig. 5A, bottom).

The last class of mitochondrial sites affected by the *orrm5* mutations with a unique response in transgenic plants is present on two clustered groups of editing sites on the *rps4* transcript. In these two groups, the effects of the full length *ORRM5* and the RRM are in opposite directions; while *ORRM5* increases the editing extent, the RRM reduces the editing extent in the transgenic plants compared with the *orrm5-3* mutant (Fig. 5B).

### Mutations in *ORRM5* do not affect the steady-state level of RNA transcripts

A possible reason for clustering of sites on the *nad3* transcript affected in the *orrm5* mutants could be an alteration in the total abundance of this transcript. A change of transcript abundance is also a possible cause of the unexpected increase of editing extents observed in the mutants. We therefore examined the transcript abundance of three transcripts that show diverse editing patterns upon *ORRM5* mutations. All the editing sites on the *nad3* transcript experience an increase of editing (see Supplementary Figs S1A and S2A). None of the sites on the *rps14* transcript are affected in the *orrm5* mutants (Supplementary Fig. S1). One out of 12 sites on the *ccmFn-2* transcript exhibit decreased editing extent in the *orrm5* mutants (Supplementary Figs S1 and S5). In all three circumstances, we did not observe any change of transcript abundance in the mutants *vs* the wild-type plants as shown in Supplementary Fig S6. We also assayed the steady-state level of *nad3*, *rps14*, and *ccmFn-2* transcripts in the transgenic lines expressing *ORRM5* or *nORRM5* in either *orrm5-2* or *orrm5-3* mutant background by RNA blots. Again we did not observe any alteration of transcript abundance (Supplementary Fig S6).

### Stable expression of *ORRM5* complements the morphological defects caused by *ORRM5* mutations

**Fig. 6.**
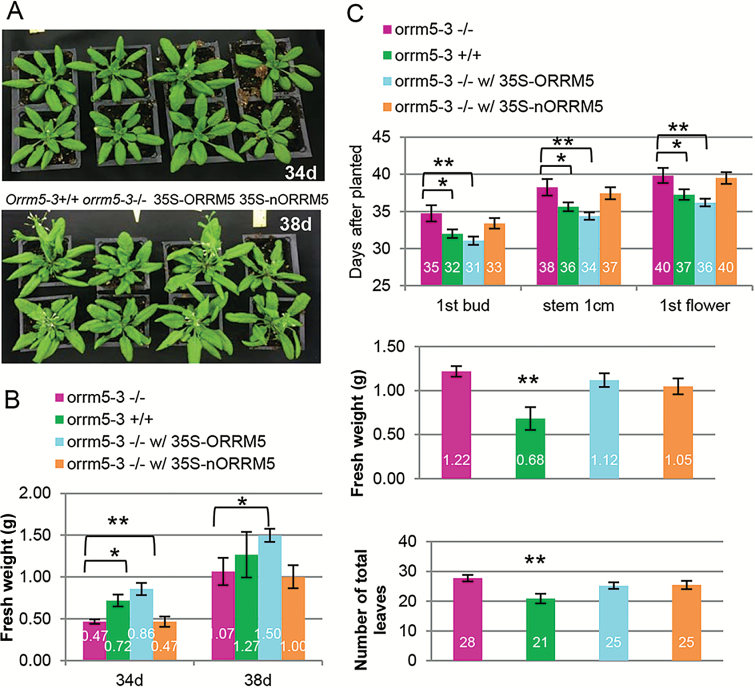
Complementation of the morphological defects in the *orrm5* mutants. *orrm5-3*+/+, wild-type siblings of *orrm5-3* mutants. *orrm5-3*–/–, *orrm5-3* homozygous mutants; 35S-ORRM5, mutant plants transformed with the coding sequence of *ORRM5* under a 35S promoter; 35S-nORRM5; mutant plants transformed with the N-terminal RRM of *ORRM5* under a 35S promoter. (A) Plant growth phenotype of plants grown at 14 h of light per day for 34 and 38 d. (B) Fresh weight of plants grown at 14 h of light per day for 34 and 38 d (*n*=4). (C) Measurement of flowering-related traits in the *orrm5* mutants, wild-type plants and the transgenic lines expressing *ORRM5* or *nORRM5*. Days taken for plants to reach these developmental stages (upper panel). 1st bud, days until visible flower buds in the center of the rosette; stem 1 cm, days until inflorescence stem reached 1 cm in height; 1st flower, days until first open flower. The fresh weight (middle panel) and the number of total leaves (lower panel) of plants at the opening of the first flower. Student’s *t*-test: *P<0.05, **P<0.01 in comparison with *orrm5-3*–/–, *n*=15. In (B, C) values represent mean±SD.

In order to assay whether the morphological defects in the *orrm5* mutants are caused by the *ORRM5* mutation, we compared the morphology of the *orrm5* homozygous mutants, their wild-type siblings, and the transgenic lines expressing the *ORRM5* or *nORRM5* transgene under the control of a 35S promoter. We planted the seeds collected from the transgenic lines used for the editing assay, and recorded the growth phenotype of the segregating T1 plants. The presence of the transgene was confirmed by genotyping. As shown in Fig. 6A, stable expression of *ORRM5* makes the plants grow faster compared with the non-transgenic *orrm5* mutants, whereas the expression of *nORRM5* does not change the morphology of the *orrm5* mutants. We also observed the complementation of the delayed growth phenotype in the transgenic lines expressing *ORRM5*, as the fresh weight of the 35S-ORRM5 transgenic lines is significantly increased compared with the non-transgenic *orrm5-3* mutants (Fig. 6B). Stable expression of *ORRM5* results in an increase of fresh weight in plants grown in long-day conditions. The fresh weight rises from 0.47 to 0.86 g on day 34 and from 1.07 to 1.50 g on day 38 (Fig. 6B). However, the expression of 35S-nORRM5 did not affect the fresh weight of the plants (Fig. 6B). We used three additional flowering time-related traits to characterize the contribution of ORRM5 to late flowering. Again, the results demonstrate that the expression of the *ORRM5* transgene complements the late flowering caused by *ORRM5* mutations, whereas *nORRM5* expression does not (Fig. 6C).

To determine whether the delayed growth phenotype is the cause of late-flowering phenotype, we recorded the fresh weight and the number of total leaves of the *orrm5* mutants, their wild-type siblings as well as the transgenic lines expressing *ORRM5* or *nORRM5*. As shown in Fig. 6B, C, *orrm5-3* mutants have higher fresh weight and total number of leaves than their wild-type siblings when their first flower opened. Given that the mutants have a greater mass than the wild-type when the first flower opens, late flowering is not the sole consequence of delayed growth in the mutants. However, the fresh weight and total number of leaves of the transgenic lines are not significantly different from the *orrm5-3* mutants (Fig. 6C) even though the complemented lines flowered at the same time as wild-type.

### *ORRM5* mutations cause reduction of cis-splicing efficiency of nad5 transcripts

Pleiotropic effects are frequently observed in mutants affected in organelle gene expression. We therefore considered whether ORRM5 might play additional roles in RNA metabolism. Chloroseq, an optimized chloroplast RNA-seq bioinformatic pipeline, has been recently developed to analyse features of chloroplast RNA metabolism including processing, editing, splicing, and relative transcript abundance (Castandet *et al.*, 2016). While this tool was built to process chloroplast RNA-seq data, we were able to adapt it to analyse our STS-PCRseq data.

**Fig. 7.**
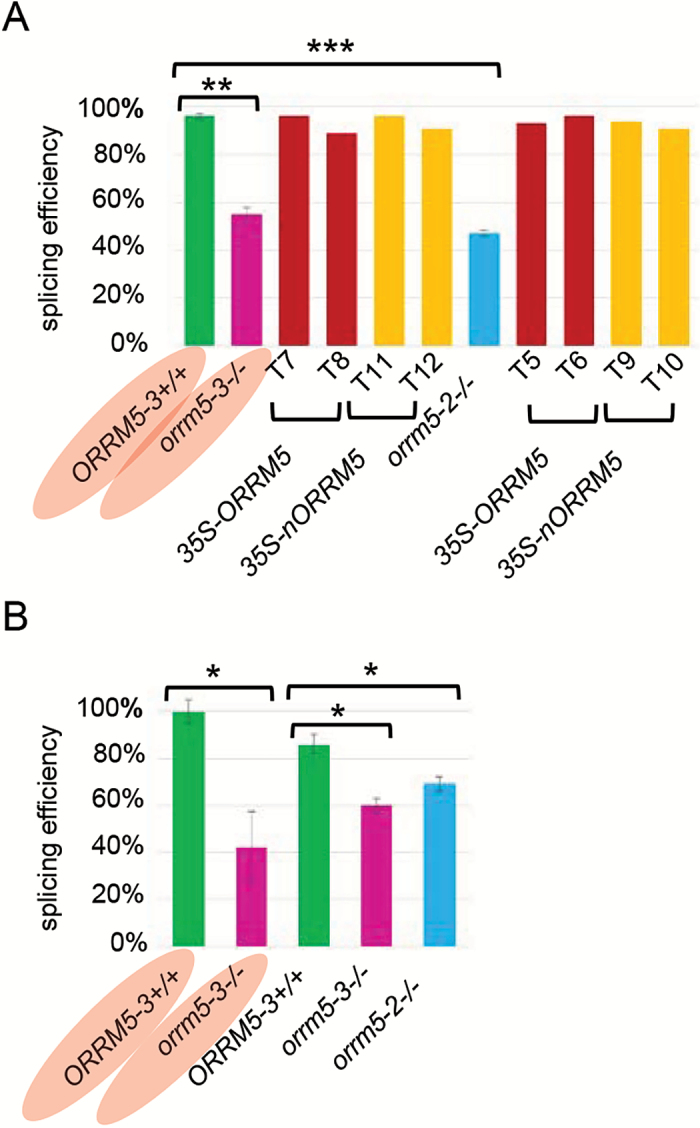
*Cis*-splicing of the first intron in the *nad5* transcript is reduced in the *orrm5* mutant plants. (A) Splicing efficiency was derived from the STS-PCRseq data by using ChloroSeq, a bioinformatic pipeline developed for chloroplast RNA-Seq. *ORRM5-3*+/+, wild-type siblings of *orrm5-3* mutants; *orrm5-3*–/–, *orrm5-3* homozygous mutants; T7 and T8, *orrm5-3*–/– w/35S:: *ORRM5*; T11 and T12, *orrm5-3*–/– w/35S:: *nORRM5*; T5 and T6, *orrm5-2*–/– w/35S:: *ORRM5*; T9 and T10, *orrm5-2*–/– w/35S:: n*ORRM5*; 35S-ORRM5, mutant plants transformed with the coding sequence of *ORRM5* under a 35S promoter; 35S-nORRM5, mutant plants transformed with the N-terminal RRM of *ORRM5* under a 35S promoter. Values represent mean±SD for *ORRM5-3*+/+, *orrm5-3–/–*, and *orrm5-2*–/–. (B) Splicing efficiency was measured by qRT-PCR with three technical replications per sample. The same RNAs for the plants highlighted in red were tested by both methods. Student’s *t*-test: *P<0.05, **P<0.01, ***P<0.001, *n*=2. Values represent mean±SD.

We analysed the known *cis*-splicing events and observed a very significant reduction of *cis*-splicing efficiency of the first intron of the *nad5* transcript in both *orrm5* mutants, as shown in Fig. 7A. Expression of *ORRM5* or *nORRM5* in either *orrm5-2* or *orrm5-3* mutant background rescues the defective *cis*-splicing of the first *nad5* intron, with the splicing efficiency increasing from ~50% to ~95% (Fig. 7A). The *nad5* transcript is composed of five exons; the maturation of the transcript requires two *cis*-splicing events to join exons 1 and 2 on the one hand and exons 4 and 5 on the other hand, and two *trans*-splicing event to join the 22-nucleotide-long exon 3 to the other two parts (see Supplementary Fig. S7) (Knoop *et al.*, 1991). Since Chloroseq was not originally developed to analyse STS-PCRseq data, we validated the defect in splicing of the first intron in *nad5* transcript by measuring splicing efficiency with a qRT-PCR assay. The same RNAs from the *ORRM5-3* wild-type and *orrm5-3* mutant plants used for the STS-PCRseq and Chloroseq analysis were assayed by qRT-PCR and showed a similar defect in splicing of *nad5* first intron (Fig. 7B). In addition, a new batch of wild-type and mutant plants were grown; the qRT-PCR assay confirmed the defect in *nad5* splicing of the first intron (Fig. 7B).

### ORRM5 interacts with ORRM2, ORRM3, and ORRM4

**Fig. 8.**
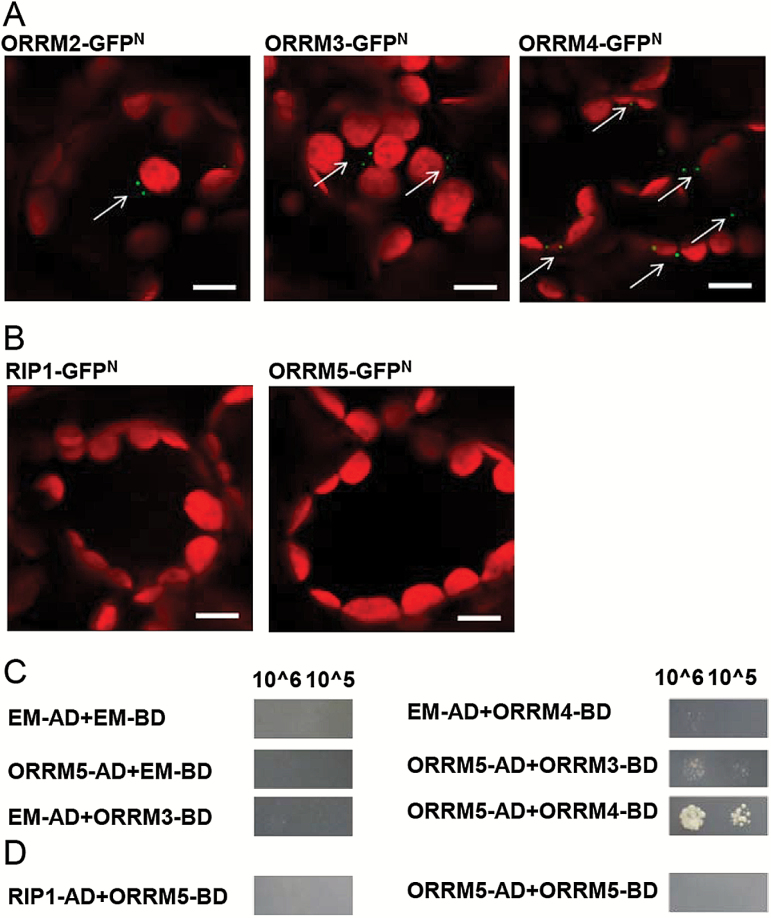
Interaction between ORRM5 and other editing factors by Y2H and BiFC assays. ORRM5-GFP^C^ has been used in all the panels shown. (A) ORRM5 interacts with ORRM2, ORRM3, and ORRM4 in BiFC assays. (B) No interactions were observed when ORRM5 was co-expressed with RIP1 or itself. Scale bar (white line) represents 10 µm. (C) ORRM5 interacts with ORRM3 and ORRM4 in the Y2H assay. (D) ORRM5 does not interact with RIP1 or itself in Y2H assays. 10^6: cells were diluted to 1 × 10^6^ cells ml^−1^ before spotted on a dropout media plate; 10^5: cells were diluted to 1 × 10^5^ cells ml^−1^ before spotted on a dropout media plate.

RNA editing is carried out by a protein complex 200–440 kDa in size (Bentolila *et al.*, 2012). ORRM5’s involvement in mitochondrial RNA editing indicates that it may interact with other components of the mitochondrial editosome. Therefore, we performed yeast two-hybrid (Y2H) and bimolecular fluorescence complementation (BiFC) assays to examine the physical interactions between ORRM5 and other editing factors. BiFC assays were performed in *Nicotiana benthamiana* by transiently coexpressing a protein fused to the N-terminal half of green fluorescent protein (GFP) (GFP^N^) with another protein fused to the C-terminal half of GFP (GFP^C^). Positive protein–protein interaction signals were observed when ORRM5-GFP^C^ was co-inoculated with ORRM2-GFP^N^, ORRM3-GFP^N^, or ORRM4-GFP^N^ (Fig. 8A). We also tested RIP1 (RNA-editing factor Interacting Protein 1), a major mitochondrial editing factor which affects editing at 474 mitochondrial C targets (Bentolila *et al.*, 2012; Bentolila *et al.*, 2013). However, no signal was observed when ORRM5-GFP^C^ was coexpressed with RIP1-GFP^N^ or ORRM5-GFP^N^ (Fig. 8B). In our Y2H assays, the predicted mature coding sequence (with the predicted transit peptide removed) was fused to the AD or BD domain. ORRM5 interacts with ORRM3 and ORRM4, but ORRM5 does not interact with RIP1 or itself in Y2H assays (Fig. 8C, D). Interaction between ORRM2 and ORRM5 cannot be determined in yeast due to auto-activation when fused to the BD domain.

## Discussion

### ORRM5 affects both RNA editing and RNA splicing

In this study, we demonstrate that ORRM5 is a mitochondrial editing factor that affects about 17% of mitochondrial sites in Arabidopsis. ORRM5 is unique in its effect on mitochondrial editing, as its absence results primarily in an increase of editing extent at the targeted C that it affects. The mutants of all the other mitochondrial factors characterized in our lab by the STS-PCRseq method show a much higher percentage of sites with decreased than increased editing extent (Table 1). Some editing sites were impaired in the mutants, but considerable editing remained. The weak effect on editing may be due to the presence of a different RRM protein that can act as an editing factor in the absence of ORRM5.

**Table 1.** Percentage of sites with altered edited extent in null mutants of mitochondrial editing factors

Mutant	Sites with increased editing extent	Sites with decreased editing extent
*rip1*	0.5%	77%
*rip3*	0.3%	26%
*orrm3*	1%	10%
*orrm4*	6%	41%
*orrm5* ^*a*^	13%	3%

^*a*^ Pooling of STS-PCRseq data from previous experiments with the current study in order to compare the effect of the different mutants on a common database resulted in a percentage of sites with an increase editing extent in *orrm5* mutant slightly different (13% and not 14%).

Like ORRM3 and ORRM4, ORRM5 carries an N-terminal RRM and a C-terminal GR motif. Previously we showed that the GR domain of ORRM4 is required for mediating its interaction with ORRM3 and itself (Shi *et al.*, 2016*b*). Another group studied NtGR-RBP1, which contains an N-terminal RRM and a C-terminal GR domain like ORRM4, and demonstrated that the GR domain is responsible for its self-association by transient interactions with its RRM (Khan *et al.*, 2014). Protein–protein interaction assays showed that ORRM3 and ORRM4 can form homodimers, whereas ORRM2 cannot (Shi *et al.*, 2015, 2016*b*). Therefore, we hypothesized the inability of ORRM2 to form homodimers to its lack of the GR motif. Surprisingly, despite the presence of a GR motif, ORRM5 does not form homodimers in our Y2H and BiFC assays. Perhaps the GR motif in ORRM5, which is much shorter than that in ORRM3 or ORRM4, has insufficient length to mediate such interactions. A short GR region could also possibly explain why ORRM5 does not interact with RIP1, whereas ORRM3 and ORRM4 do.

One possible explanation for the action of ORRM5 on mitochondrial editing extent is sequestration of other *bona fide* editing factors such as ORRM2, ORRM3, and ORRM4, preventing them from fulfilling their role. In this scenario, in *orrm5* mutants, the absence of ORRM5 releases the other ORRM mitochondrial editing factors, resulting in an increase of editing extent of the sites under their control. This hypothesis is strongly supported by the significant dependence of the editing extent in *orrm5* mutant and in *orrm2* or *orrm*3 mutants (*P*<10^–7^, Supplementary Fig. S8). The large value of the χ^2^ is mostly due to the excess of observed sites experiencing both an increase of editing extent in *orrm5* mutants and a decrease of editing extent in either *orrm2* or *orrm3* mutants (see Supplementary Fig. S8).

In the transgenic lines overexpressing *ORRM5*, numerous mitochondrial sites that were invariant in the mutant lines exhibit a significant alteration of their editing extents. Among these, a majority, 27% *vs* 6% in *orrm5-3* transgenic plants and 15% *vs* 6% in *orrm5-2* transgenic plants, show a decrease of editing extent *vs* an increase of editing extent (see Supplementary Fig. S4). This result supports the inhibitory effect of ORRM5 on the mitochondrial editing extent. A possible explanation is that increasing chaperone activity by using 35S overexpression causes more changes in RNA structure and thus potentially in RNA editing. However, an analysis of the mitochondrial sites showing a decrease of their editing extent in the transgenic lines together with the effect of other mitochondrial editing factors, ORRM2 or ORRM3, shows a strong dependency relationship (Supplementary Fig. S9). The large value of the χ^2^ is mostly due to the excess of observed sites experiencing both a decrease of editing extent in *orrm5* transgenic lines and a decrease of editing extent in either *orrm2* silenced or *orrm3* mutant plants (Supplementary Fig. S9). Taken together, these results support a model in which an excess of ORRM5 in the transgenic lines might sequester more mitochondrial editing factors than in the wild-type, thus decreasing preferentially the editing extent of the sites under the control of the sequestered editing factors.

Our results indicate that the RRM of ORRM5 is sufficient for ORRM5’s function in RNA editing, at least for the majority of the sites that are increased in editing extent in the mutant. Complementation of editing defects by the RRM alone was also true in the cases of ORRM1, ORRM3, and ORRM4 (Shi *et al.*, 2015, 2016*b*). We also demonstrate that the RRM of ORRM5 is essential for efficient *cis*-splicing of the first intron on the *nad5* transcript. However, the expression of the RRM could not compensate the morphological defects caused by the *orrm5* mutants. We previously found that the expression of the GR domain of ORRM4 complements the late flowering phenotype in the *orrm4* mutants (Shi *et al.* 2016*b*). Taken together, these results indicate that the GR domain may participate in ORRM5’s role in plant growth and development. Given that the RRM could only rescue the molecular defects rather than the physiological changes in *orrm5* mutants, it is quite possible that the physiological changes observed in *orrm5* mutants are not caused by its defects in RNA processing. However, how ORRM5 influence plant growth and development is not yet identified.

*ORRM5* mutations cause both RNA editing and RNA splicing defects. These processes both occur post-transcriptionally in mitochondria of flowering plants. Several studies have addressed the question of the temporal relationship of RNA editing and RNA splicing. Partially edited spliced transcripts were observed (e.g. *cox2*, *nad1*), indicating that editing is not a strict prerequisite for splicing (Sutton *et al.*, 1991; Yang and Mulligan, 1991; Gray and Covello, 1993). On the other hand, unspliced transcripts or mRNA intermediates were found to be less edited than spliced transcripts on average, indicating that the temporal relationship of splicing and editing is not entirely independent (Gualberto *et al.*, 1991; Sutton *et al.*, 1991; Yang and Mulligan, 1991; Gray and Covello, 1993). Other studies demonstrated that these two RNA processing events may be coupled in some circumstances so that one event is a prerequisite for the other. Intron RNA editing is essential for *nad1*, *rps10*, and *mat-r-nad1e-nad5c* transcript splicing in plant mitochondria (Borner *et al.*, 1995; Castandet *et al.*, 2010; Farré *et al.*, 2012), whereas the spinach *ndhA* site 1 is edited in intronless transcripts but remains unedited in unspliced transcripts when expressed in tobacco (Schmitz-Linneweber *et al.*, 2001). The reduction in splicing efficiency of *nad5* first intron in *orrm5* mutants is moderate when compared with the defects observed in other mutants of *nad5* splicing factors such as PPR proteins TANG2 and OTP43 (Colas des Francs-Small *et al.*, 2014). The moderate effect could be due to redundancy with another RRM protein or other splicing factor that can partially replace ORRM5’s function in a splicing complex. Alternatively, the reduced splicing could be due to a pleiotropic effect.

A number of plant RRM proteins are involved in RNA splicing. An organellar RRM protein, pentatricopeptide repeat protein 4 (PPR4), which contains both an RRM and a PPR domain, is required for *rps12 trans*-splicing in maize (*Zea mays*) and consequently for the accumulation of plastid ribosomes (Schmitz-Linneweber *et al.*, 2006). Serine/arginine-rich (SR) proteins play critical roles in nuclear alternative pre-mRNA splicing (Zahler *et al.*, 1993). SR proteins carry one or two RRMs and an RS domain (Palusa *et al.*, 2007). The RRM is required for RNA binding, whereas the RS region is necessary for interacting with other protein partners (Palusa *et al.*, 2007). Additionally, organellar RRM-containing proteins CP31A and CP29A are involved in multiple chloroplast RNA processes, including RNA splicing (Tillich *et al.*, 2009; Kupsch *et al.*, 2012). The loss of CP33A, another RRM-containing protein, causes change of splicing efficiency of a few chloroplast transcripts. CP33A is required for mRNA accumulation, particularly relevant for unspliced and precursor mRNAs (Teubner *et al.*, 2016). The higher degree of sensitivity of unspliced transcripts to loss of CP33A may mimic the splicing defects caused by the *cp33a* mutation (Teubner *et al.*, 2016).

### ORRM5 participates in plant stress responses

ORRM5 was first identified as a mitochondrial RNA-binding protein named mitochondrial RNA-binding protein 1a (At-mRBP1a; Vermel *et al.*, 2002). The potato homolog to At-mRBP1a was isolated by its affinity to ssDNA in purified potato mitochondria. ORRM5/At-mRBP1a has a much higher affinity to poly (U) than to other three homo-ribopolymers or to DNA, and its expression could be induced by cold treatment, but not by wounding, drought or ABA treatment (Vermel *et al.*, 2002). ORRM5 was also characterized as glycine-rich RNA-binding protein 2 (GRP2), glycine-rich RNA binding protein 2 (GR-RBP2) or RNA-binding glycine-rich subclass A 5 (RBGA5) (Lorković and Barta, 2002; Kim *et al.*, 2007*b*; Krishnamurthy *et al.*, 2015). It has been reported to affect seed germination of Arabidopsis plants under salt stress, accelerate seed germination and seedling growth under cold stress, and enhance the cold tolerance in Arabidopsis plants (Kim *et al.*, 2007*b*). In another study, expression of the Arabidopsis *ORRM5*/*GRP2* in rice (*Oryza sativa*) was able to improve rice grain yield under drought stress conditions (Yang *et al.*, 2014). Additionally, ORRM5/GRP2 complements the cold sensitivity of an *Escherichia coli* BX04 mutant and exhibits transcription anti-termination activity, indicating that it has an RNA chaperone activity during the cold adaptation process (Kim *et al.*, 2007*a*; Kim *et al.*, 2007*b*). The anti-termination activity requires melting of an RNA secondary structure in *E. coli*. If ORRM5 can similarly alter RNA secondary structure in mitochondria, *orrm5* mutation may lead to changes in RNA structure that might cause indirect effects in RNA editing/splicing. Alternatively, ORRM5 may perform multiple functions depending on the environment, acting as RNA chaperone under stress conditions while functioning as an RNA editing/splicing factor under normal conditions. What role ORRM5 plays in stress response is not evident from our analysis of its functions in RNA editing and splicing.

Results from several studies indicate that stress conditions, such as exposure to heat, cold, or heavy metals, could affect the efficiency or patterns of RNA splicing and/or RNA editing (Luehrsen *et al.*, 1994; Simpson and Filipowicz, 1996). CP31A, a chloroplast ribonucleoprotein that protects plant against cold stress, is involved in the splicing of *ndhB* and *ycf3* mRNAs under cold stress (Kupsch *et al.*, 2012). Additionally, a cold stress-upregulated nuclear protein, STABILIZED1 (STA1), contributes to the splicing of transcripts encoded by the *COR15A* gene in cold-treated Arabidopsis plants (Lee *et al.*, 2006). Results from a recent study that analysed published RNA-Seq datasets derived from Arabidopsis grown under stress conditions demonstrated that heat stress results in a global reduction in splicing and editing efficiency in Arabidopsis chloroplast (Castandet *et al.*, 2016). RNA editing and splicing of the wheat mitochondrial *cox2* transcript were also impaired under low-temperature conditions (Kurihara-Yonemoto and Kubo, 2010).

Several organelle RNA editing factors have been reported to participate in plant stress responses. A member of the Arabidopsis PPR protein family, MEF11/LOI1, participates in mitochondrial RNA editing as well as ABA and stress responses (Sechet *et al.*, 2015). ORRM1, the founder member of the ORRM family, is essential for chloroplast RNA editing in Arabidopsis and maize (Sun *et al.*, 2013). A recent study reports that ORRM1 is involved in cold stress tolerance in Arabidopsis (Wang *et al.*, 2016). The expression of mitochondrial RNA editing factor *ORRM3*/*GR-RBP3* could also be induced by cold treatment. ORRM5 is relevant to cold and drought stress responses as discussed earlier (Kim *et al.*, 2007*a*; Kim *et al.*, 2007*b*; Yang *et al.*, 2014). However, the association between stress tolerance and RNA splicing and/or RNA editing awaits further investigation.

## Supplementary data

Supplementary data are available at *JXB* online.

Dataset S1. Number of reads at each editing site for each plant assayed by STS-PCRseq.

Fig. S1. Percentage of affected edited sites/transcript in the *orrm5* mutants.

Fig. S2. Editing on the *nad3* and *rps4* transcripts is affected by *ORRM5* mutation.

Fig. S3. Distribution of the complementation effect in the transgenic lines transformed with the full length *ORRM5*.

Fig. S4. Distribution and behavior of the editing sites that did not show any significant change in the mutant lines (*vs* the wild-type) in the transgenic lines transformed with the full length *ORRM5*.

Fig. S5. Editing defect at the *ccmFn-2* C320 site in the *orrm5* mutants.

Fig. S6. Examples of transcript abundance in mutant, wild-type, and transgenic lines.

Fig. S7. Representation of the complex structure of the *nad5* transcript.

Fig. S8. Contingency tables of the number of mitochondrial sites experiencing editing change in *orrm* mutants.

Fig. S9. Contingency tables of the number of mitochondrial sites experiencing editing decreases in transgenic lines overexpressing *ORRM5* and in *orrm*2 silenced or *orrm3* mutants.

Table S1. Primers used in this study.

## Supplementary Material

supplementary_figures_S1_S9

supplementary_table_S1

supplementary_dataset_S1
