# Supplementary material for: ORRM5, an RNA recognition motif-containing protein, has a unique effect on mitochondrial RNA editing
Source: J Exp Bot. 2017 May 26;68(11):2833–47. doi: 10.1093/jxb/erx139 (PMC5853588; doi:10.1093/jxb/erx139)
Supplement: supplementary_figures_S1_S9 [file erx139_suppl_supplementary_figures_s1_s9.pdf]

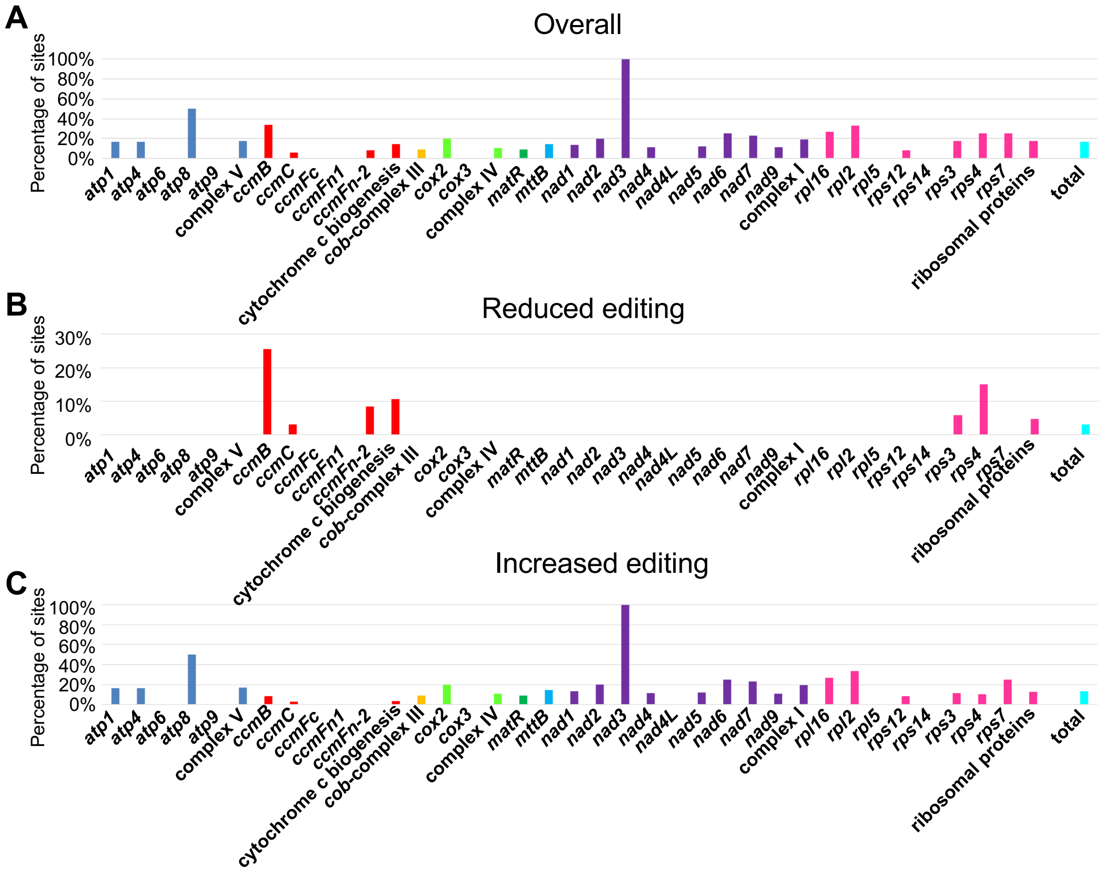

Supplementary Figure S1. Percentage of affected edited sites/transcript in the *orrm5* mutants. **(A)** Percentage of sites affected by *ORRM5* mutations on each transcript. Each bar represents a transcript color-coded according to the complex to which it belongs. **(B)** Percentage of sites experiencing reduced editing extents by *ORRM5* mutations on each transcript. **(C)** Percentage of sites showing increased editing efficiency on each transcript as a result of *orrm5* mutations.

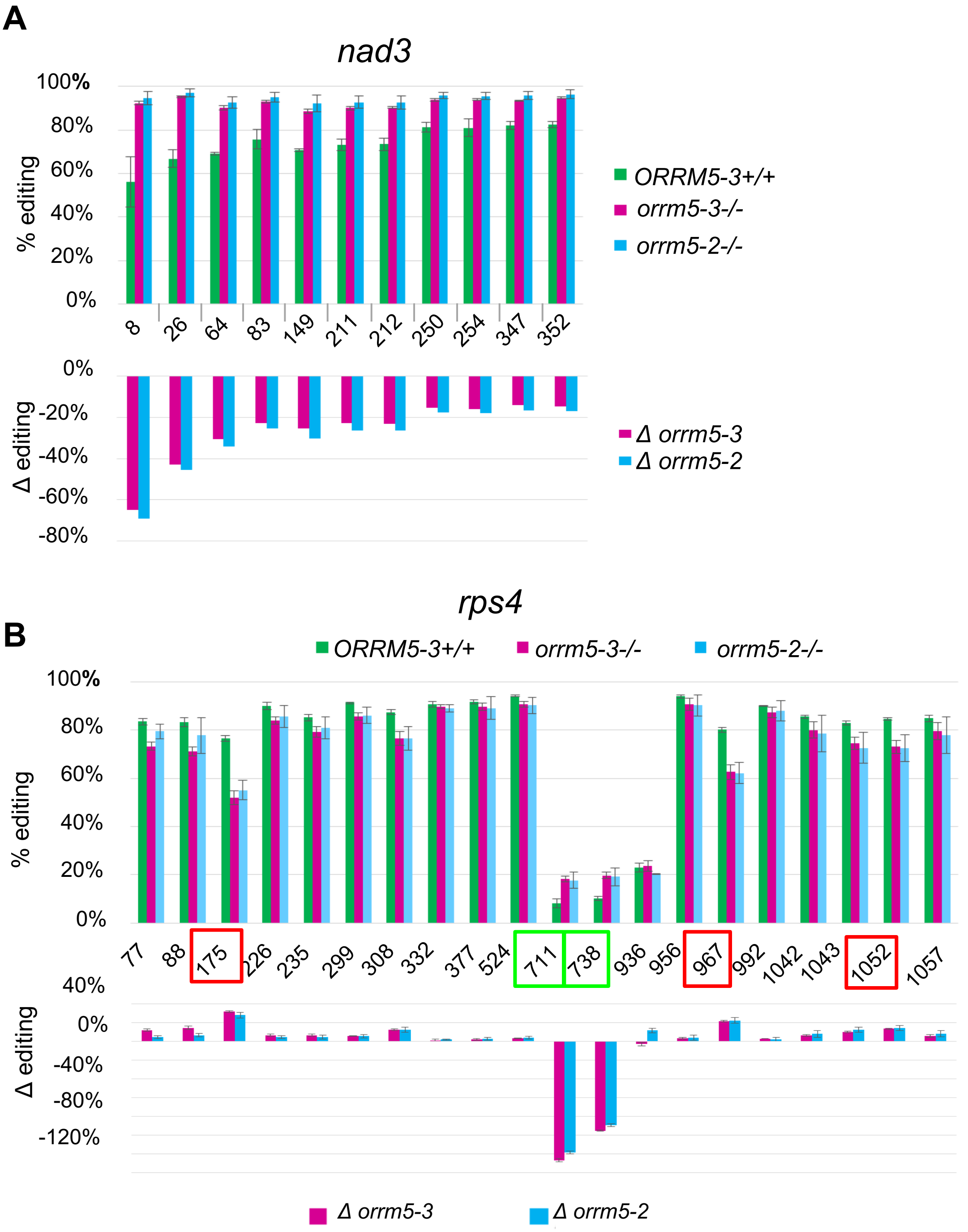

Supplementary Figure S2. Editing on the *nad3* and *rps4* transcripts is affected by *ORRM5* mutation. *ORRM5-3*+/+, wild-type siblings of *orrm5-3* mutants; *orrm5-3* -/-, *orrm5-3* homozygous mutants; *orrm5-2*-/-, *orrm5-2* homozygous mutants (n=2). **(A)** *ORRM5* mutations result in increased editing extent at all the mitochondrial C targets on the *nad3* transcript. X-axis represents position of different editing sites on the *nad3* transcript. The lower panel represents the variation in editing extent (Δ editing); Δ*orrm5-2*, (% editing of *ORRM5-3*+/+ minus % editing of *orrm5-2*-/-)/ % editing of *ORRM5-3*+/+; Δ*orrm5-3*, (% editing of *ORRM5-3*+/+ minus % editing of *orrm5-3*-/-)/ % editing of *ORRM5-3*+/+. **(B)** Effects of *ORRM5* mutations on mitochondrial RNA editing on the *rps4* transcript. Like in Figure 4A, the upper panel represents editing extent at different sites on the transcript with the location on the X axis. Sites experiencing a significant reduction of editing extents ($\Delta \geq 10\%$, $P < 1.6e\text{-}6$) are in red squares, whereas sites showing an increase of editing extents are in green squares ($\Delta \geq 10\%$, $P < 1.6e\text{-}6$). The lower panel represents the variation in editing extent (Δ editing) with Δ*orrm5-2* and Δ*orrm5-3* calculated as in Figure 4A.

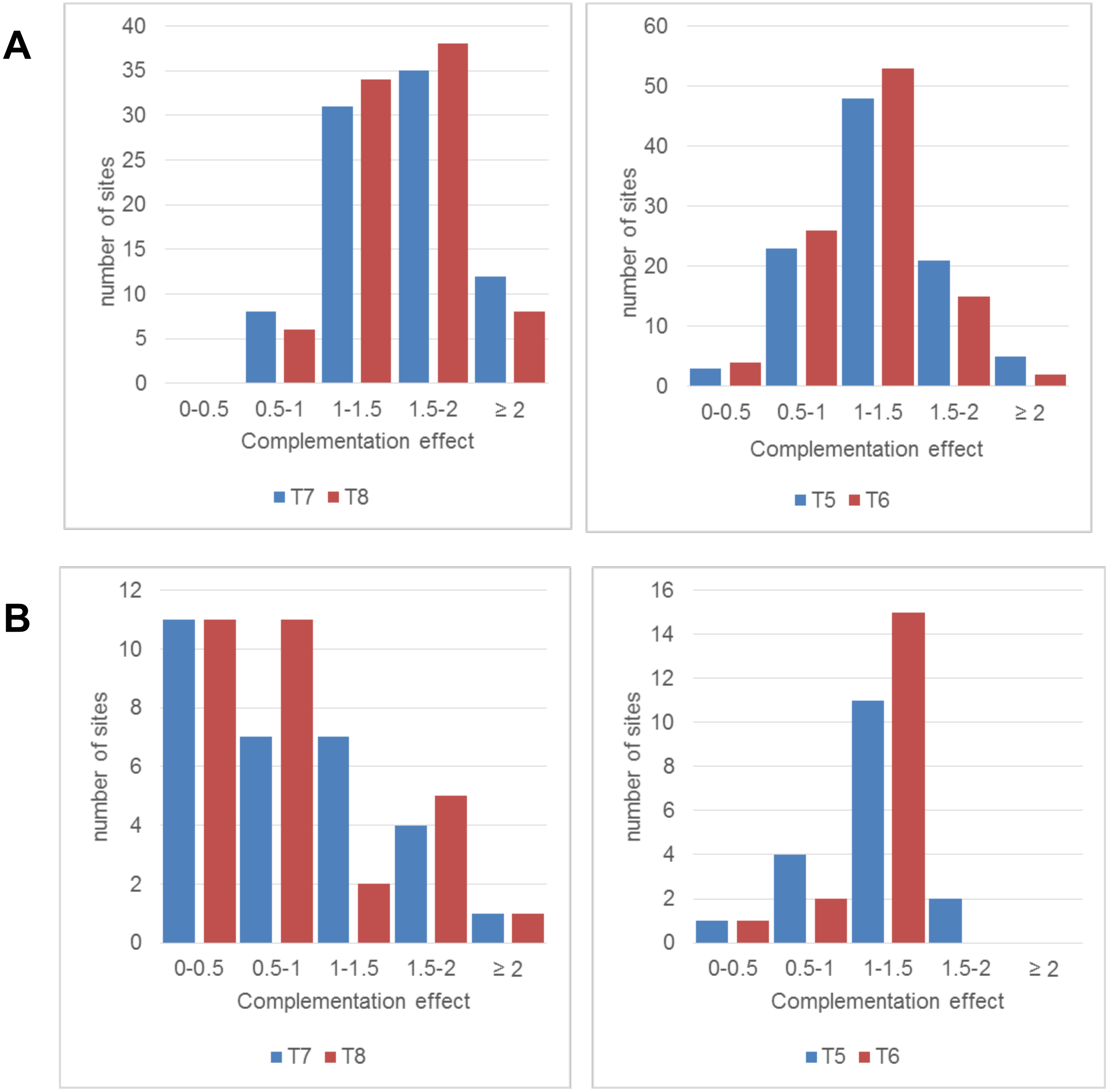

Supplementary Figure S3. Distribution of the editing sites that showed a significant change of editing extent in the mutant lines (vs. the wild-type) in the transgenic lines transformed with the full length *ORRM5* according to the complementation effect. The complementation effect is calculated as (% editing in transgenic-% editing in mutant)/(% editing in wild-type-% editing in mutant). The complementation effect is a measure of the response observed in the transgenic lines in relation to the one observed in the wild-type; when this metric equals 1 the response in the transgenic is similar to the one observed in the wild-type. **(A)** Distribution for the sites that were significantly increased in the mutant plants**. (B)** Distribution for the sites that were significantly decreased in the mutant plants. T7 and T8, *orrm5-3* transgenic lines, T5 and T6, *orrm5-2* transgenic lines.

A

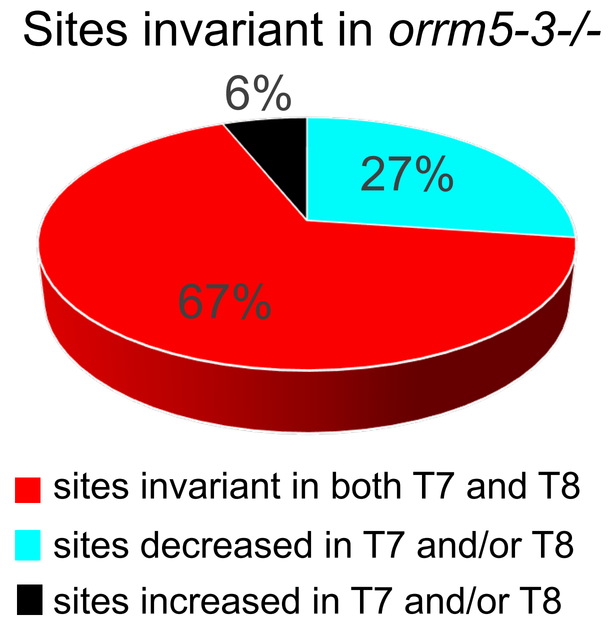

B

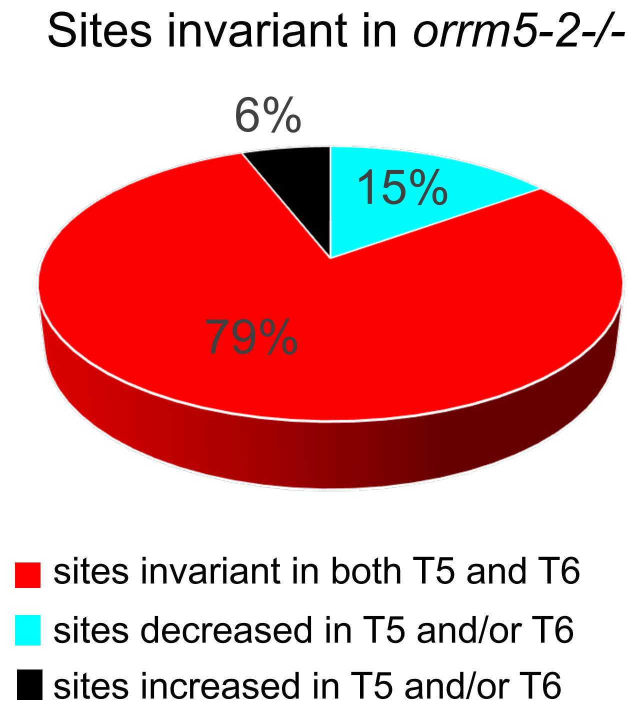

Supplementary Figure S4. Distribution and behavior of the editing sites that did not show any significant change in the mutant lines (vs. the wild-type) in the transgenic lines transformed with the full length *ORRM5*. **(A)** Distribution in T7 and T8, *orrm5-3* transgenic lines**. (B)** Distribution in T5 and T6, *orrm5-2* transgenic lines.

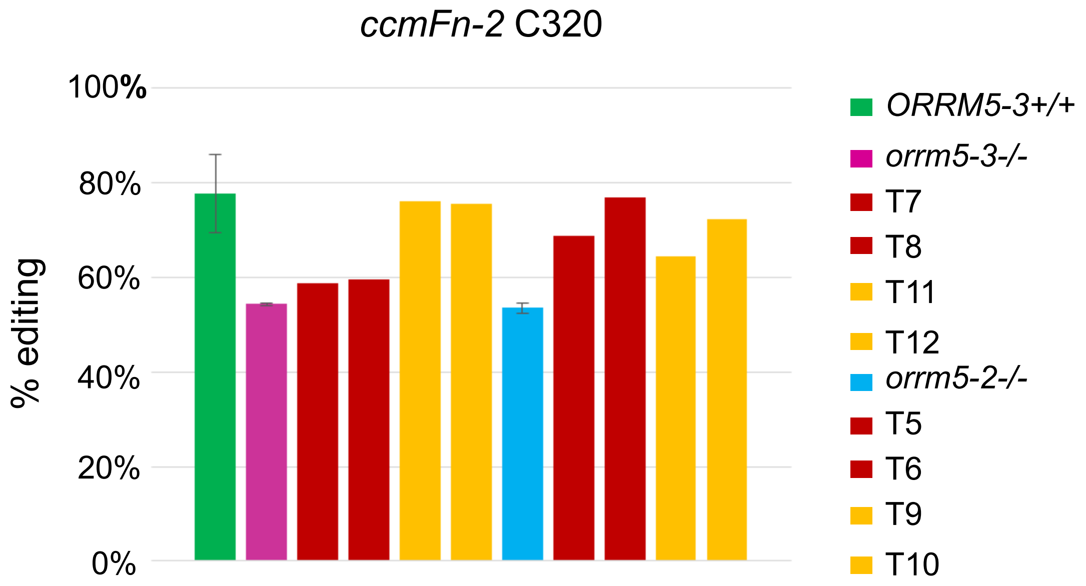

Supplementary Figure S5. Editing defect at the *ccmFn-2* C320 site in the *orrm5* mutants and its complementation by expressing *ORRM5* or the N-terminal RRM domain of *ORRM5* (*nORRM5*). Editing extents were measured by STS-PCRseq. T7 and T8: *orrm5-3*-/- w/35S:: *ORRM5*; T11 and T12: *orrm5-3*-/- w/35S:: *nORRM5*; T5 and T6: *orrm5-2*-/- w/35S:: *ORRM5*; T9 and T10: *orrm5-2*-/- w/35S:: *nORRM5*. Both *orrm5-3* and *orrm5-2* mutants show a decrease of editing extent compared to the *ORRM5-3* wild-type. In *orrm5-3* transgenic plants, only the RRM (T11, T12) is able to complement the editing defect to wild-type level. In *orrm5-2* transgenic plants, both the full length *ORRM5* (T5, T6) and the RRM (T9, T10) are able to complement the editing defect to wild-type level or lower. Values represent mean ± SD for *ORRM5-3*+/+, *orrm5-3*-/-, and *orrm5-2*-/- (n=2).

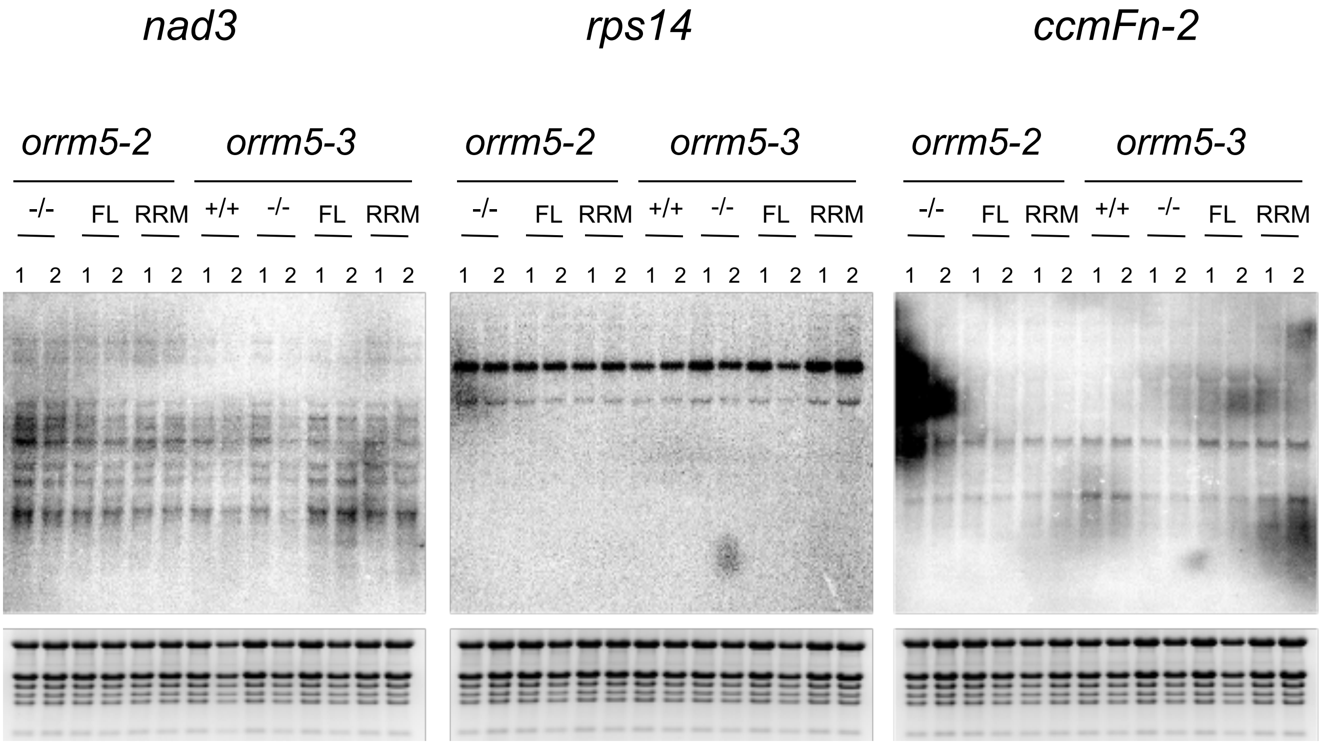

Supplementary Figure S6. Transcript abundance of *nad3*, *rps14* and *ccmFn-2* in the *orrm5* mutants versus the controls examined by RNA blots. *orrm5-2*, plants in an *orrm5-2* homozygous mutant background; *orrm5-3*, plants in an *orrm5-3* homozygous mutant background; +/+, wild-type siblings of *orrm5-3* mutants. -/-, mutant plants that do not contain the *ORRM5* or *nORRM5* transgene; FL, mutant plants transformed with the coding sequence of *ORRM5* under a 35S promoter; RRM, mutant plants transformed with the N-terminal RRM motif of *ORRM5* under a 35S promoter. The negative of the ethidium bromide staining gel in the bottom panel serves as a loading control.

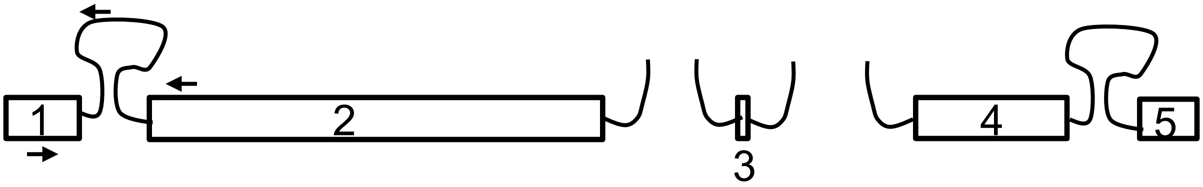

Supplementary Figure S7. Representation of the complex structure of the *nad5* transcript. The five exons are symbolized by squares drawn to scale and numbered 1 to 5. The introns are sketched as solid lines. The maturation of *nad5* transcript necessitates two *cis*-splicing events to join exon1 and exon2 and exon4 and exon5. In addition, two *trans*-splicing events are needed to join exon3 to exon2 and exon4. The arrows represent the primers used in the qRT-PCR assay to determine the splicing efficiency of intron1.

	orrm5 up	*orrm5* other	
orrm2 down	16<br>*5.13*<br>(23.03)	23<br>*33.87*<br>(3.49)	39
orrm2 other	57<br>*67.87*<br>(1.74)	459<br>*448.13*<br>(0.26)	516
	73	482	555

χ^2 = 28.528 *P* = 9 e-8

	orrm5 up	*orrm5* other	
orrm3 down	21<br>*7.63*<br>(23.44)	37<br>*50.37*<br>(3.55)	58
orrm3 other	52<br>*65.37*<br>(2.73)	445<br>*431.63*<br>(0.41)	497
	73	482	555

χ^2 = 30.134 *P* = 4 e-8

	orrm5 up	*orrm5* other	
orrm4 down	19<br>*29.73*<br>(3.87)	207<br>*196.27*<br>(0.59)	226
orrm4 other	54<br>*43.27*<br>(2.66)	275<br>*285.73*<br>(0.40)	329
	73	482	555

χ^2 = 7.518 *P* = 6 e-3

Supplementary Figure S8. Contingency tables of the number of mitochondrial sites experiencing an increase in *orrm5* mutant (*orrm5* up) or no increase in *orrm5* mutant (*orrm5* other) and a decrease in other mitochondrial editing factors (*orrm2*, *orrm3*, or *orrm4* down) or no decrease in these mutants or silenced tissues (*orrm2*, *orrm3*, or *orrm4* other). The results with *orrm2* were obtained on silenced tissues. The observed number of sites are in plain character while the expected values are in italics, and the individual χ^2 are displayed in parentheses. Below each contingency table is given the χ^2 value for a test of independence and the p value. The total number of mitochondrial sites commonly surveyed between this study and the previous studies on ORRM2. ORRM3, and ORRM4 is 555.

A

	T7,T8 down	T7,T8 other	
orrm2 down	15<br>*5.54*<br>(16.13)	6<br>*15.46*<br>(5.79)	21
orrm2 other	103<br>*112.46*<br>(0.80)	323<br>*313.54*<br>(0.29)	426
	118	329	447

χ^2 = 23 *P* = 2 e-6

	T7,T8 down	T7,T8 other	
orrm3 down	28<br>*9.5*<br>(36)	8<br>*26.5*<br>(12.9)	36
orrm3 other	90<br>*108.5*<br>(3.15)	321<br>*302.5*<br>(1.13)	411
	118	329	447

χ^2 = 53.2 *P* = 0

B

	T5,T6 down	T5,T6 other	
orrm2 down	11<br>*2.74*<br>(24.88)	9<br>*17.26*<br>(3.95)	20
orrm2 other	50<br>*58.26*<br>(1.17)	375<br>*366.74*<br>(0.19)	425
	61	384	445

χ^2 = 30.19 *P* = 4 e-8

	T5,T6 down	T5,T6 other	
orrm3 down	22<br>*4.25*<br>(74.15)	9<br>26.75<br>(11.78)	31
orrm3 other	39<br>*56.75*<br>(5.55)	375<br>*357.25*<br>(0.88)	414
	61	384	445

χ^2 = 92.36 *P* = 0

Supplementary Figure S9. Contingency tables of the number of mitochondrial sites experiencing either a decrease in transgenic lines, (T7, T8 down) or (T5, T6 down), or no decrease in transgenic lines, (T7, T8 other) or (T5, T6 other), and a decrease in other mitochondrial editing factors (*orrm2* or *orrm3* down) or no decrease in these mutants or silenced tissues (*orrm2*, *orrm3* other). The results with *orrm2* were obtained on silenced tissues. This analysis is restricted to the invariant sites that did not show significant change in the mutant plants either *orrm5-3*, or *orrm5-2* when compared to the wild-type. The observed number of sites are in plain character while the expected values are in italics, and the individual χ^2 are displayed in parentheses. Below each contingency table is given the χ^2 value for a test of independence and the p value. **(A)** Contingency tables obtained with *orrm5-3* transgenic lines. **(B)** Contingency tables obtained with *orrm5-2* transgenic lines
